# Piezoelectric Shunt Stiffness in Rhombic Piezoelectric Stack Transducer with Hybrid Negative-Impedance Shunts: Theoretical Modeling and Stability Analysis

**DOI:** 10.3390/s19153387

**Published:** 2019-08-01

**Authors:** Leiying He, Wenguang Zheng, Chenxue Zhao, Chuanyu Wu

**Affiliations:** 1 Faculty of Mechanical Engineering & Automation, Zhejiang Sci-Tech University, Hangzhou 310018, China; 2 School of Mechanical Engineering & Automation, University of Science and Technology Liaoning, Anshan 114051, China

**Keywords:** controlled stiffness, negative capacitance, shunt damping, vibration control, piezoelectric

## Abstract

Negative-capacitance shunted piezoelectric polymer was investigated in depth due to its considerable damping effect. This paper discusses the novel controlled stiffness performance from a rhombic piezoelectric stack transducer with three hybrid negative-impedance shunts, namely, negative capacitance in series with resistance, negative capacitance in parallel with resistance, and negative inductance/negative capacitance (NINC) in series with resistance. An analytical framework for establishing the model of the coupled system is presented. Piezoelectric shunt stiffness (PSS) and piezoelectric shunt damping (PSD) are proposed to analyze the stiffness and damping performances of the hybrid shunts. Theoretical analysis proves that the PSS can produce both positive and negative stiffness by changing the negative capacitance and adjustable resistance. The Routh–Hurwitz criterion and the root locus method are utilized to judge the stability of the three hybrid shunts. The results point out that the negative capacitance should be selected carefully to sustain the stability and to achieve the negative stiffness effect of the transducer. Furthermore, negative capacitance in parallel with resistance has a considerably better stiffness bandwidth and damping performance than the other two shunts. This study demonstrates a novel electrically controlled stiffness method for vibration control engineering.

## 1. Introduction

Piezoelectric transducers are widely used for vibration control [1,2,3], energy harvesting [4,5,6,7,8], health monitoring [9,10], and sensing. Piezoelectric shunt damping involves the connection of an electrical impedance to terminals of a piezoelectric transducer (PZT), and it was widely studied since Forward carried out a preliminary experimental demonstration of the feasibility of using external electronic circuits to control mechanical vibrations in optical systems [11]. Hagood and von Flotow [12] studied a resistive shunt that is able to dissipate vibrational energy in the form of heat. A piezoelectric patch that consists of a single resonant circuit with an inductor can generate electrical resonance to reduce vibration [13,14,15]. The passive multimode resonant shunts, such as the Hollkamp shunt [16], the current-blocking shunt [17], the current-flowing shunt [18], and the series–parallel shunt [19], were investigated to control multimodal vibrations of host structures. These resonant shunts were applied to control the vibration of a compact disc read-only memory (CD-ROM) [20], a hard disk drive (HDD) disk–spindle system [21,22,23], a smart panel [24], a uniform bimorph beam (simulating chatter) [25], etc. The closed-form solution [26], H∞ [27], matrix inequalities [28], and equal modal damping [29] were employed to optimize the shunt parameters. The pure passive resistive shunt offers a little damping to the mechatronic system. The resonant shunt works like a tuned mass damper that is sensitive to the change in natural frequency [30]. To overcome these two drawbacks of the passive shunt, adaptive multimode resonant shunts were proposed to adjust the change in frequency so as to achieve considerable vibration control [31]. 

Compared to those shunts that only use passive electronics, such as a resistor, a capacitor, and an inductor, the active shunt needs an external electrical energy to drive it, such as operational amplifiers, transistors, and electronic switches [2,3,32,33]. Fleming et al. [34] used the active Linear-Quadratic-Gaussian (LQG), H2, and H∞ methods to optimize a suitable impedance of the shunt. The piezoelectric transducer can be electrically simplified to a capacitance and a current source. The negative capacitance is able to cancel the inherent capacitance of the piezoelectric transducer so as to realize broadband vibration control [35]. The negative capacitance can be constructed by a negative impedance converter (NIC) that can also be used to construct negative resistance [36,37,38,39] and negative inductance [40]. Neubauer et al. [41] studied the effect of negative-capacitance shunted piezoelectric transducers in absorbing systems. Manzoni et al. [42] discussed the values of the electric elements composing the negative capacitance to improve vibration reduction efficiency and to avoid instability at low frequencies. Beck et al. [43] suggested that the negative-capacitance shunt can modify the effective modulus of the piezoelectric transducer, and discussed the power output and efficiency of negative capacitance. Han et al. [44] proposed an adaptive shunt that consists of a switched inductance/resistance in parallel with negative capacitance to improve damping performance. Neubauer et al. [45] used it to control the vibration of a bladed disc. Gripp et al. [46] described an adaptive resonant piezoelectric vibration absorber enhanced by synthetic negative capacitance. The resonant shunt circuit autonomously adapted the inductance value by comparing the phase difference of the vibration velocity and the current flowing through the shunt. Synthetic negative capacitance was employed to enhance the vibration attenuation performance. Pohl et al. [47] studied an adaptive negative-capacitance circuit to improve the robustness of PSD, and to improve the performance of the negative-capacitance shunt by enlarging the output voltage to the requirements of piezoelectric transducers. The other studies and applications on negative-capacitance shunts can be found in Reference [48]. 

The existing research efforts mainly focused on how to use negative-capacitance shunted piezoelectric patches to suppress structural vibrations [49,50]. Preumont [51] and Marneffe [1] began applying the negative-capacitance shunted piezoelectric stack to the vibration control of a truss structure. Manzoni et al. [42] and Beck et al. [43] showed that a carefully chosen negative capacitance can produce a change in stiffness and the frequency shift phenomenon. Heuss [52] discussed tuning of a vibration absorber with negative-capacitance shunted piezoelectric patches. That is the first time the value of negative capacitance used was bigger than the inherent capacitance of a transducer. These studies mainly discussed the damping performance of PSD.

Up to now, few studies considered the stiffness effect of negative-capacitance shunted PSD. Zheng et al. [53] discussed the tuning of natural frequency with an electromagnetic shunt mass. Consequently, this study focuses on the stiffness mechanism of PSD, and discusses the use of the negative stiffness to enhance the structural vibration isolation performance. The aim of the study is different from the previous studies [49,50,51,52]. The theoretical model of the coupled electromechanical system is obtained according to Lagrange’s equation. The piezoelectric shunt stiffness is proposed and analyzed. The stability of the transducer is theoretically analyzed according to the Routh–Hurwitz criterion and graphically discussed with the root locus method. The damping and stiffness effects are also discussed.

## 2. Materials and Methods

### 2.1. Modeling of the Rhombic Piezoelectric Stack Transducer

**Figure 1:**
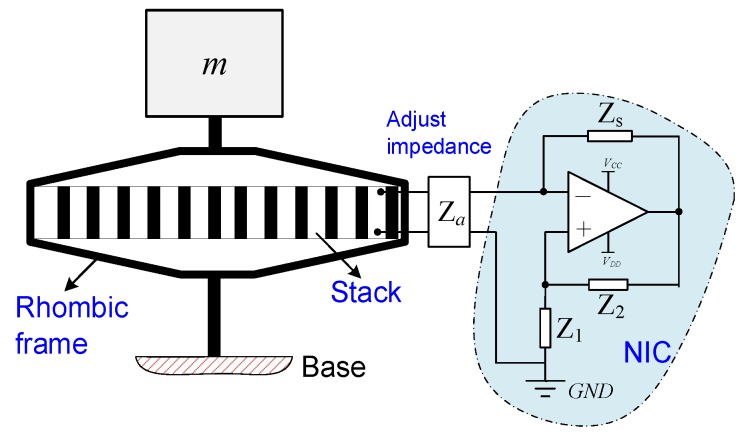
Model of a rhombic piezoelectric stack transducer with a hybrid negative-impedance shunt circuit. *Z_a_* is the adjustable impedance of shunt; it can be in series and parallel forms. *Z*_1_, *Z*_2_, and *Z_s_* are utilized to construct the different types of shunt.

Figure 1 shows a rhombic piezoelectric stack transducer with a hybrid negative-impedance shunt circuit. Compared to positive impedance, the equivalent value of the negative impedance is negative. The rhombic frame can magnify the output displacement of the piezoelectric transducer like an operation amplifier; the corresponding amplification coefficient can be found in Reference [54]. The terminals of the piezoelectric stack transducer connect to a hybrid NIC shunt, which consists of an adjustable impedance *Z_a_* and an NIC. The NIC is constructed by an operational amplifier [1,35,55]. The input impedance of the circuit created by the passive impedances *Z*_1_, *Z*_2_, and *Z_s_* is $Z=-\frac{Z_{1}}{Z_{2}}Z_{s}$ [43]. If $Z_{1}=Z_{2}$, the equivalent impedance of the operational amplifier will be $-Z_{s}$. When *Z_s_* is replaced with a capacitor, an inductor, or a resistor, we can obtain equivalent negative capacitance, negative inductance, or negative resistance. In the present study, we discuss the effect of negative capacitance on the stiffness performance of PSD. It should be noted that the rhombic frame is used to install the stack. In general, the stiffness of the rhombic frame is very big; thus, it should be carefully designed for vibration isolation application. 

**Figure 2:**
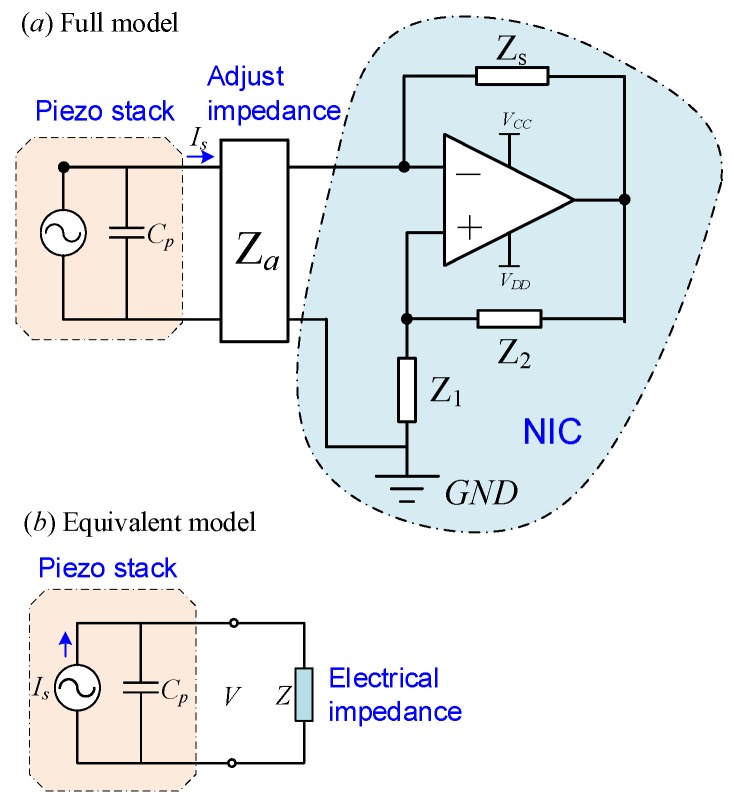
Electrical model of the piezoelectric stack transducer with the hybrid shunt: (**a**) full schematic, and (**b**) equivalent impedance model.

Figure 2a is the electrical equivalent model of the piezoelectric stack transducer with the hybrid shunt. The governing equation of the rhombic piezoelectric stack transducer with the hybrid negative-impedance shunt is modeled with Lagrange’s equation,

(1) $$\frac{d}{dt}\left(\frac{\partial L}{\partial {\dot{q}}_{i}}\right)-\frac{\partial L}{\partial q_{i}}=Q_{i},$$

where *L* = *T* + *W* − *U* is the Lagrangian of the system, and *Q_i_* is the nonconservative force associated with the generalized coordinates. *T* is the kinetic energy of the piezoelectric transducer,

(2) $$T=\frac{1}{2}{\dot{x}}^{T}m\dot{x}.$$

*U* is the restored deformation energy of the rhombic frame,

(3) $$U=\frac{1}{2}x^{T}kx,$$

where *k* is the stiffness of the rhombic frame. 

*W* is the energy of the piezoelectric stack transducer,

(4) $$W=\frac{1}{2}C_{P}V^{2}+nd_{33}K_{a}Vx-\frac{1}{2}K_{a}x^{2},$$

where $C_{p}=C(1-k_{p}^{2})$ is the capacitance of the transducer under constant extension, and the electromechanical coupling factor of the transducer $k_{p}^{2}$ is the efficiency of the conversion of mechanical energy into electricity, which ranges around 0.5 for PZT [1]. The stack includes *n* discs. The stiffness with short-circuited electrodes is *K_a_*, and *d*_33_ is the piezoelectric constant. V is the voltage between the electrodes. Figure 2b is the equivalent model of hybrid shunts. The nonconservative virtual work is related to the damping of the rhombic frame. The dissipated work through the shunt circuit and the external excitation force is as follows:

(5) $$\delta W_{nc}=-c\dot{x}\delta x-I_{s}\delta V+F\delta x.$$

Substituting Equations (2)–(5) into Equation (1), the governing equations of the coupled electromechanical system are as follows:

(6) $$m\ddot{x}+c\dot{x}+(k+K_{a})x-\theta V=F,$$

(7) $$C_{P}\dot{V}+\theta \dot{x}+I_{s}=0,$$

where *θ* = *nd*_33_*K_a_* is the electromechanical coupling coefficient of the stack, and *I_s_* is the current flowing in the circuit. The structural damping coefficient *c* is represented by *c* = 2*ζω_n_*/*m*, and the structural damping ratio *ζ* can be selected between 0.5% and 1%. The natural frequency *ω_n_* is represented by $\sqrt{(k+K_{a})/m}$.

### 2.2. Concept of Controlled Stiffness with Hybrid Negative-Impedance Shunts

#### 2.2.1. Model for the Negative-Impedance Shunt

In the time domain, Equations (6) and (7) cannot reflect the stiffness effect of the hybrid shunt. In the Fourier domain, these two equations are as follows:

(8) $$-\left[(k+K_{a})-m\omega^{2}+jc\omega \right]x-\theta V=F,$$

(9) $$j\omega C_{P}V+j\theta \omega x+I_{s}=0.$$

If the shunt is represented by an equivalent impedance *Z* shown in Figure 2b, we have *I_s_* = *V*/*Z*. Then, Equation (9) becomes

(10) $$V=\frac{-j\omega \theta }{j\omega C_{p}+1/Z}x.$$

Substituting Equation (10) into Equation (8), the transfer function is as follows:

(11) $$\frac{x}{F}=\frac{1}{(k+K_{a})-m\omega^{2}+j\omega \left(c+c_{sh}\right)},$$

(12) $$c_{sh}=\frac{\theta^{2}}{j\omega C_{p}+1/Z}.$$

It can be found that the shunt brings the damping into the piezoelectric stack transducer, which can possibly reduce structural vibration. If Equation (12) is written as

(13) $$c_{sh}=\frac{1-j\omega ZC_{p}}{1+\omega^{2}Z^{2}C_{p}^{2}}\theta^{2}Z,$$

then Equation (11) is reorganized as

(14) $$\frac{x}{F}=\frac{1}{(k+K_{a})-(m+m_{s})\omega^{2}+j\omega \left(c+c_{s}\right)},$$

(15) $$m_{s}=\frac{-\theta^{2}Z^{2}C_{p}}{1+\omega^{2}Z^{2}C_{p}^{2}},$$

(16) $$c_{s}=\frac{\theta^{2}Z}{1+\omega^{2}Z^{2}C_{p}^{2}}.$$

These three equations imply that the introducing of a shunt circuit brings both the damping and the mass effects into the transducer, where *m_s_* and *c_s_* are defined as the piezoelectric shunt mass (PSM) and piezoelectric shunt damping, respectively. The variation of the mass and stiffness influences the natural frequency of the transducer. Therefore, Equation (14) can also be reorganized as

(17) $$\frac{x}{F}=\frac{1}{(k+K_{a}+k_{s})-m\omega^{2}+j\omega \left(c+c_{s}\right)},$$

(18) $$k_{s}=\frac{\omega^{2}\theta^{2}Z^{2}C_{p}}{1+\omega^{2}Z^{2}C_{p}^{2}},$$

where *k_s_* is defined as the piezoelectric shunt stiffness. Equation (18) suggests that the PSS is associated with the excitation frequency, the adjustable impedance, and the capacitance of the shunt circuit.

**Figure 3:**
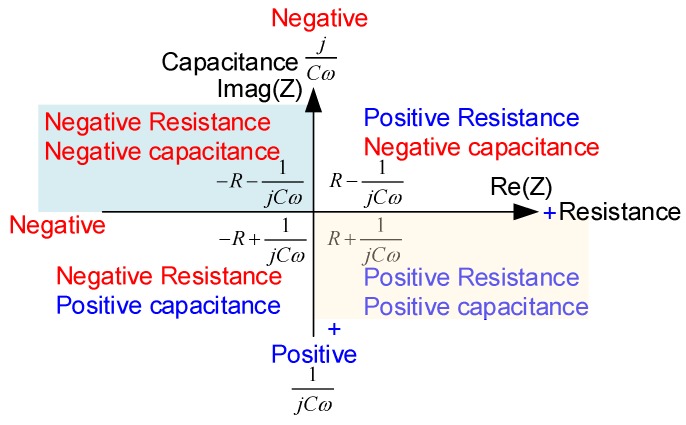
Complex plot of capacitance considering both the positive and negative cases.

Figure 3 presents the positive and negative capacitance at the complex plane. The horizontal axis represents the resistance, and the vertical axis represents the capacitance. The controlled PSS will be different upon changing the shunt impedance in different quadrants. The negative capacitance −1/*jcω* can be rewritten as *j*/*cω*; it is somewhat like the positive inductance, but the frequency relationship is reciprocal. In previous studies [42,51], the negative capacitance was laid at the first quadrant. In this study, we move the impedance location of the shunt to the second quadrant, and discuss the stiffness and damping effects by changing the value of the negative capacitance.

#### 2.2.2. Negative Capacitance in Series with Resistance

**Figure 4:**
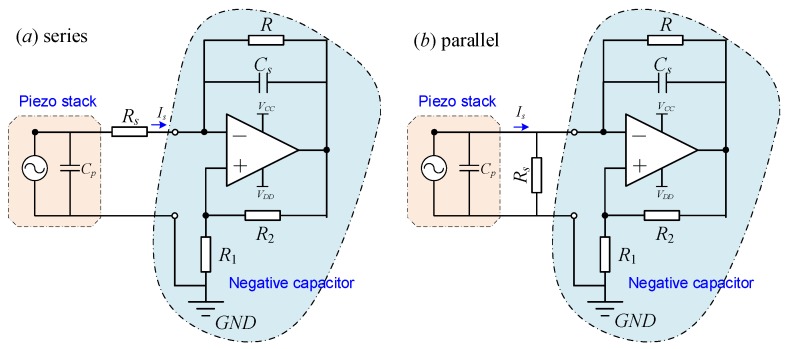
Piezoelectric stack transducer with negative capacitance and adjustable resistance *R_s_*: (**a**) in series and (**b**) in parallel. The resistor *R* is utilized to increase the leakage of the capacitor *C_s_*.

A resistor *R* connected in series with a capacitor *C_s_* can increase the leakage of the negative resistance, as shown in Figure 4a. *R* is necessary and should be large enough due to the bias currents flowing from the non-ideal operational amplifier. The parallel resistor and capacitor act like a high-pass filter allowing bias current to flow to ground, thus preventing the capacitor from acquiring a direct current (DC) charge [35]. The equivalent impedance of the NIC circuit is

(19) $$Z_{s}=-\Gamma_{R}\frac{1}{1/R+j\omega C_{s}},$$

where Γ*_R_* = *R*_1_/*R*_2_. Assuming that Γ*_R_* = 1 and taking the adjustable resistor *R_s_* into consideration, the total impedance of this series shunt is

(20) $$Z=R_{s}-\frac{1}{1/R+j\omega C_{s}}.$$

If this hybrid shunt connects to the piezoelectric stack transducer, then Equation (15) becomes

(21) $$c_{sh}=c_{s}-j\frac{k_{s}}{\omega }.$$

This equation shows the relationship between the PSS and PSD. The coefficients *c_s_* and *k_s_* are as follows:

(22) $$c_{s}=\theta^{2}\frac{\left(1/R-\omega^{2}R_{s}C_{s}C_{p}\right)\left(R_{s}/R-1\right)+\left(C_{s}-C_{p}+R_{s}C_{p}/R\right)\omega^{2}R_{s}C_{s}}{{\left(1/R-\omega^{2}R_{s}C_{s}C_{p}\right)}^{2}+{\left(C_{s}-C_{p}+R_{s}C_{p}/R\right)}^{2}\omega^{2}},$$

(23) $$k_{s}=-\frac{\theta^{2}\omega^{2}\left[R_{s}C_{s}\left(1/R-\omega^{2}R_{s}C_{s}C_{p}\right)-\left(R_{s}/R-1\right)\left(C_{s}-C_{p}+R_{s}C_{p}/R\right)\right]}{{\left(1/R-\omega^{2}R_{s}C_{s}C_{p}\right)}^{2}+{\left(C_{s}-C_{p}+R_{s}C_{p}/R\right)}^{2}\omega^{2}}.$$

#### 2.2.3. Negative Capacitance in Parallel with Resistance

Figure 4b is the schematic of the negative capacitance in parallel with *R_s_*, where the total impedance of the shunt is

(24) $$Z=\frac{1}{1/R_{s}-(1/R+j\omega C_{s})}.$$

Substituting into Equation (21) and simplifying it, we can obtain $c_{s}$ and $k_{s}$.

(25) $$c_{s}=\frac{\theta^{2}\left(1/R_{s}-1/R\right)}{{\left(1/R_{s}-1/R\right)}^{2}+\omega^{2}{\left(C_{p}-C_{s}\right)}^{2}},$$

(26) $$k_{s}=\frac{\theta^{2}\omega^{2}\left(C_{p}-C_{s}\right)}{{\left(1/R_{s}-1/R\right)}^{2}+\omega^{2}{\left(C_{p}-C_{s}\right)}^{2}}.$$

#### 2.2.4. Negative Inductance and Negative Capacitance in Series with Resistance

**Figure 5:**
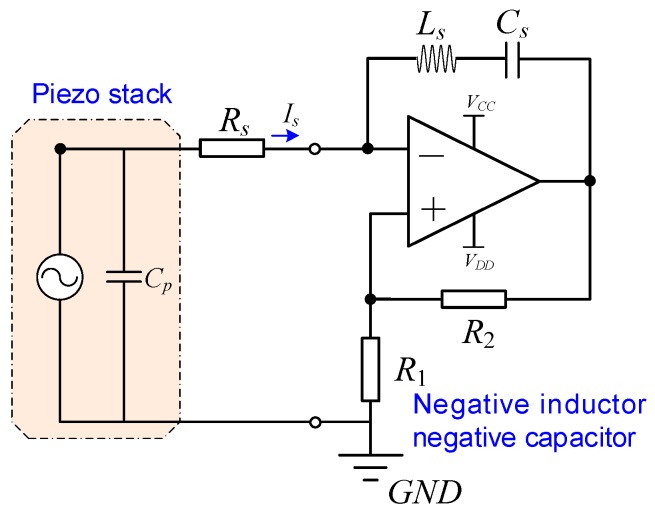
Schematic of the piezoelectric stack transducer with the negative inductance/negative capacitance (NINC) in series with the resistance shunt circuit.

If the equivalent impedance *Z_s_* in Figure 2a is replaced by an inductor *L_s_* and a capacitor *C_s_* in series, and *Z*_1_ and *Z*_2_ are resistors, the schematic is as presented in Figure 5. Assuming that *R*_1_ is equal to *R*_2_, then the impedance of the NIC is

(27) $$Z_{s}=-\Gamma_{R}\left(j\omega L_{s}+\frac{1}{j\omega C_{s}}\right).$$

Thus,

(28) $$Z=R_{s}-\Gamma_{R}\left(j\omega L_{s}+\frac{1}{j\omega C_{s}}\right).$$

Substituting Equation (28) into Equation (12), one can obtain

(29) $$c_{s}=\theta^{2}\frac{R_{s}C_{s}C_{p}\Gamma_{R}\left(1-\omega^{2}L_{s}C_{s}\right)+R_{s}C_{s}\left\{C_{s}-C_{p}\left[\Gamma_{R}\left(1-\omega^{2}L_{s}C_{s}\right)\right]\right\}}{{\left(\omega R_{s}C_{s}C_{p}\right)}^{2}+{\left\{C_{s}-C_{p}\left[\Gamma_{R}\left(1-\omega^{2}L_{s}C_{s}\right)\right]\right\}}^{2}},$$

(30) $$k_{s}=-\frac{\theta^{2}\left[\left\{C_{s}-C_{p}\left[\Gamma_{R}\left(1-\omega^{2}L_{s}C_{s}\right)\right]\right\}\Gamma_{R}\left(1-\omega^{2}L_{s}C_{s}\right)-\omega^{2}R_{s}^{2}C_{s}^{2}C_{p}\right]}{{\left(\omega R_{s}C_{s}C_{p}\right)}^{2}+{\left\{C_{s}-C_{p}\left[\Gamma_{R}\left(1-\omega^{2}L_{s}C_{s}\right)\right]\right\}}^{2}}.$$

## 3. Results

### 3.1. Analysis of the PSS for the Three Hybrid Shunts

We already obtained the PSS *k_s_* for the three hybrid shunts. The influence of PSD on PSS is quite important, which determines the design of the controlled stiffness transducer. If *k_s_* is divided by *c_s_*,

(31) $$\kappa_{c}=\frac{k_{s}}{c_{s}},$$

$\kappa_{c}$ is defined as the stiffness and damping ratio of the PSD.

For the negative capacitance in series with the resistance shunt case, according to Equation (31),

(32) $$\kappa_{c}=-\omega^{2}\frac{R_{s}C_{s}\left(1/R-\omega^{2}R_{s}C_{s}C_{p}\right)-\left(R_{s}/R-1\right)\left(C_{s}-C_{p}+R_{s}C_{p}/R\right)}{\left(1/R-\omega^{2}R_{s}C_{s}C_{p}\right)\left(R_{s}/R-1\right)+\left(C_{s}-C_{p}+R_{s}C_{p}/R\right)\omega^{2}R_{s}C_{s}}.$$

For the negative capacitance in parallel with the positive resistance shunt case,

(33) $$\kappa_{c}=\omega^{2}\frac{C_{p}-C_{s}}{1/R_{s}-1/R}.$$

For the negative inductance and negative capacitance in series with resistance case,

(34) $$\kappa_{c}=\frac{\left\{C_{s}-C_{p}\left[\Gamma_{R}\left(1-\omega^{2}L_{s}C_{s}\right)\right]\right\}\Gamma_{R}\left(1-\omega^{2}L_{s}C_{s}\right)-\omega^{2}R_{s}^{2}C_{s}^{2}C_{p}}{R_{s}C_{s}C_{p}\Gamma_{R}\left(1-\omega^{2}L_{s}C_{s}\right)+R_{s}C_{s}\left\{C_{s}-C_{p}\left[\Gamma_{R}\left(1-\omega^{2}L_{s}C_{s}\right)\right]\right\}}.$$

Table 1 lists the parameters of the piezoelectric stack transducer and the hybrid shunts that are obtained from the experiment. According to the theoretical model of the PSS and PSD obtained in Section 3, the stiffness performance of the hybrid shunts is discussed below.

**Figure 6:**
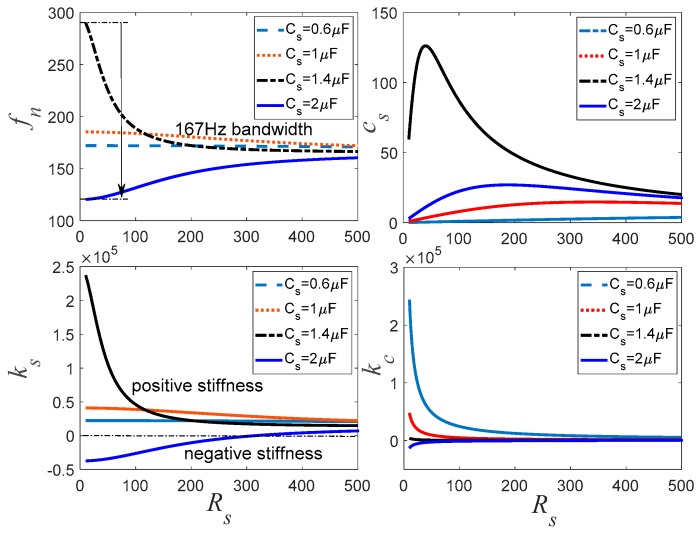
Controlled stiffness analysis for the negative capacitance in series with *R_s_* shunt.

Figure 6 shows the variation of the natural frequency *f_n_*, *k_s_*, *c_s_*, and *κ_c_* with respect to the adjustable resistance *R_s_* for the negative capacitance in series with *R_s_* shunt. When *C_s_* is −0.6 μF, the changes of *f_n_* and *k_s_* are very small, which means that it is hard to generate the electrically controlled stiffness effect. The corresponding damping effect is also small. When the negative capacitance is −1 μF, *f_n_* and *k_s_* increase. When the negative capacitance increases to −1.4 μF, which means that the absolute value of negative capacitance approximates to the inherent capacitance of the piezoelectric stack *C_p_*, then *f_n_* and PSS begin to change in a very large range. The stiffness is a positive value that increases the natural frequency of the transducer. In this case, the damping effect changes with the change of *R_s_*, and it can easily find an optimal value. When the negative capacitance is further increased to −2 μF, which means *C_s_* is bigger than *C_p_*, *f_n_* also decreases apparently. In this case, the PSS produces the negative stiffness effect that decreases with the increase of *R_s_*. The corresponding damping effect is within an acceptable range.

**Figure 7:**
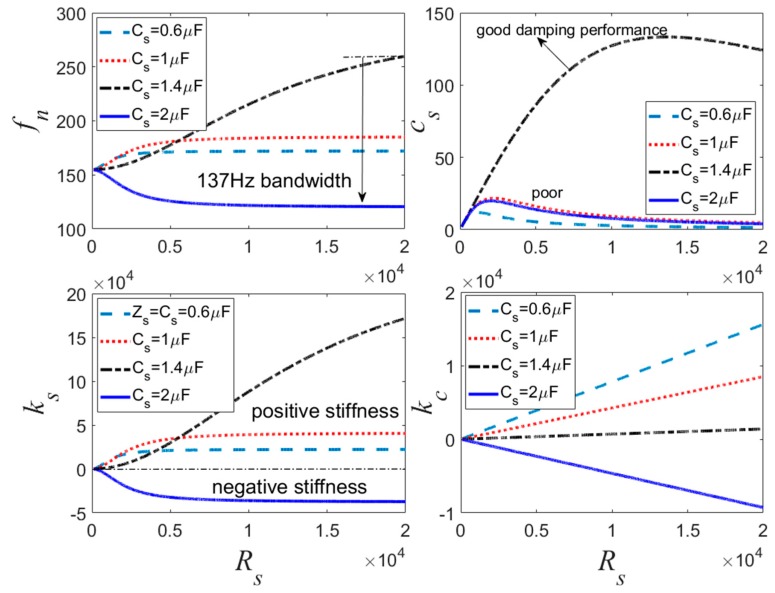
Controlled stiffness analysis for the negative capacitance in parallel with *R_s_* shunt.

Figure 7 is the variation of the natural frequency *f_n_*, *k_s_*, *c_s_*, and *κ_c_* with respect to the adjustable resistance *R_s_* for the negative capacitance in parallel with resistance shunt. The negative capacitance for −0.6 μF, −1 μF, −1.4 μF, and −2 μF cases is discussed. When the negative capacitance is increased from −0.6 μF to −1.4 μF, *k_s_* is positive, increasing the natural frequency of the transducer, and the natural frequency also increases with the increase of the negative capacitance. The damping performance is excellent when the negative capacitance is −1.4 μF. When the negative capacitance is further increased to −2 μF, *f_n_* decreases dramatically. In this case, the PSS produces the negative stiffness effect that decreases with the increase of *R_s_*. 

**Figure 8:**
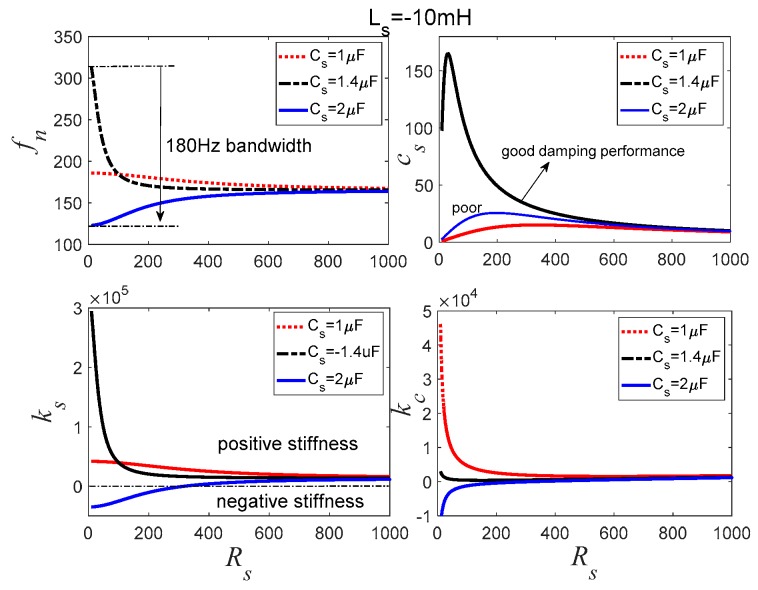
Controlled stiffness analysis for the negative inductance/negative capacitance in series with *R_s_* shunt.

Figure 8 is the variation of the natural frequency, PSS, PSD, and *κ_c_* with respect to the resistance *R_s_* for the negative inductance/negative capacitance in series with resistance shunt. When the negative capacitance is −1 μF, the changes of *f_n_* and *k_s_* are small, and the *c_s_* is also small, making it hard to control the vibration of the system. When the negative capacitance increases to −1.4 μF, *f_n_* and *k_s_* begin to change in a very large range. *k_s_* is positive, increasing the natural frequency of the transducer. The PSD *c_s_* increases apparently and the optimal *c_s_* appears when *R_s_* is 165.2 Ω. When the negative capacitance is further increased to −2 μF, *f_n_* also decreases like the other two kinds of shunts; *k_s_* is also a negative stiffness.

Figure 6, Figure 7 and Figure 8 also imply that *κ_c_* is very big when *c_s_* is small. When *c_s_* increases, *κ_c_* tends to a small value. This demonstrates that *κ_c_* can be used for evaluating the damping effect of the PSD. If we combine *f_n_* and *k_s_* curves shown in Figure 6, Figure 7 and Figure 8 together, it can be found that the negative capacitance in parallel with resistance case has a relative stable controlled natural frequency and better stiffness performance compared to the other two hybrid shunts. With this hybrid shunt, *κ_c_* curves are straight lines. When the absolute value of negative capacitance is bigger than *C_p_*, the controlled stiffness may be negative, which results in the decrease of the natural frequency of the transducer. Conversely, when the absolute value of negative capacitance is smaller than *C_p_*, the controlled stiffness is positive, which increases the natural frequency of the transducer. *k_s_* increases with the increase of the negative capacitance of the shunt. The natural frequency is determined mostly by the negative capacitance, and the PSS *c_s_* is determined by *R_s_*. However, when the absolute value of the negative capacitance approximates to *C_p_*, *R_s_* dramatically influences *k_s_*. Consequently, the negative capacitance and the adjustable resistance should be carefully selected to sustain considerable stiffness and damping performance.

### 3.2. Stability Analysis

#### 3.2.1. Routh–Hurwitz Criterion

1. Negative capacitance in series with resistance

Note that when *s* = *jω*, $\omega_{n}^{2}=(k+K_{a})/m$, then the characteristic function of the piezoelectric stack transducer with the hybrid shunts can be obtained according to Equation (11),

(35) $$s^{2}+\frac{c+c_{sh}}{m}s+\omega_{n}^{2}=\;0.$$

For the negative capacitance in series with resistance shunt, when *R* → ∞, then

(36) $$c_{sh}=\frac{\theta^{2}\left(sR_{s}C_{s}-1\right)}{s^{2}R_{s}C_{s}C_{p}-s\left(C_{p}-C_{s}\right)}.$$

The inherent capacitance of the piezoelectric stack *C_p_* and the negative capacitance *C_s_* are all in the microfarad scale; thus, *C_s_* *C_p_* can be neglected to some extent, and the characteristic function of the closed-loop system is as follows:

(37) $$\left(C_{p}-C_{s}\right)s^{2}+\left[2\varsigma \omega_{n}\left(C_{p}-C_{s}\right)-\theta^{2}R_{s}C_{s}/m\right]s+\left[\theta^{2}/m+\omega_{n}^{2}\left(C_{p}-C_{s}\right)\right]=0.$$

The Routh array is

(38) $$\begin{array}{c} s^{2}\\ s^{1}\\ s^{0} \end{array}|\begin{array}{cc} C_{p}-C_{s} & \theta^{2}/m+\omega_{n}^{2}\left(C_{p}-C_{s}\right)\\ 2\varsigma \omega_{n}\left(C_{p}-C_{s}\right)-\theta^{2}R_{s}C_{s}/m & 0\\ \theta^{2}/m+\omega_{n}^{2}\left(C_{p}-C_{s}\right) & 0 \end{array}.$$

The necessary and sufficient condition for the stability of this system is that the first column of the Routh array in Equation (38) is positive. 

When *C_p_* > *C_s_*, the following relationship is required to keep the stability of the control system:

(39) $$2\varsigma \omega_{n}\left(C_{p}-C_{s}\right)-\theta^{2}R_{s}C_{s}/m>0;\theta^{2}/m+\omega_{n}^{2}\left(C_{p}-C_{s}\right)>0.$$

Then, one can get

(40) $$C_{s}<\min\left\{\frac{C_{p}}{1+\theta^{2}R_{s}/\left(2\varsigma m\omega_{n}\right)},C_{p}+\frac{\theta^{2}}{m\omega_{n}^{2}}\right\}.$$

The abovementioned equation suggests that

(41) $$\frac{C_{p}}{1+\theta^{2}R_{s}/\left(2\varsigma m\omega_{n}\right)}<C_{p}<\frac{\theta^{2}}{m\omega_{n}^{2}}+C_{p}.$$

Thus, *C_s_* should be selected as

(42) $$C_{s}<\frac{C_{p}}{1+\theta^{2}R_{s}/\left(2\varsigma m\omega_{n}\right)}.$$

When *C_p_* < *C*_s_, the capacitance of the circuit is negative; with the same process, the following condition should be met:

(43) $$C_{s}>\max\left\{\frac{2\varsigma \omega_{n}C_{p}}{1+\theta^{2}R_{s}/(2\varsigma \omega_{n}m)},C_{p}+\frac{\theta^{2}}{m\omega_{n}^{2}}\right\}.$$

Then, we have

(44) $$C_{s}>C_{p}+\frac{\theta^{2}}{m\omega_{n}^{2}}.$$

2. Negative capacitance in parallel with positive resistance

For the negative capacitance in parallel with resistance case, when *R* → ∞, the characteristic function is as follows:

(45) $$a_{3}s^{3}+a_{2}s^{2}+a_{1}s+a_{0}=0,$$

(46) $$\begin{array}{l} a_{3}=R_{s}\left(C_{p}-C_{s}\right)\\ a_{2}=2\varsigma \omega_{n}R_{s}\left(C_{p}-C_{s}\right)+1\\ a_{1}=2\varsigma \omega_{n}+\omega_{n}^{2}R_{s}\left(C_{p}-C_{s}\right)+\frac{\theta^{2}R_{s}}{m}\\ a_{0}=\omega_{n}^{2} \end{array},$$

(47) $$\begin{array}{c} s^{3}\\ s^{2}\\ s^{1}\\ s^{0} \end{array}|\begin{array}{cc} a_{3} & a_{1}\\ a_{2} & a_{0}\\ b_{1} & 0\\ a_{0} & 0 \end{array}.$$

Thus, the system should meet the following conditions:

(48) $$\begin{array}{l} 2\varsigma \omega_{n}R_{s}\left(C_{p}-C_{s}\right)+1>0\\ \frac{\left[2\varsigma \omega_{n}R_{s}\left(C_{p}-C_{s}\right)+1\right]\left[2\varsigma \omega_{n}+\omega_{n}^{2}R_{s}\left(C_{p}-C_{s}\right)+\frac{\theta^{2}R_{s}}{m}\right]-\omega_{n}^{2}R_{s}\left(C_{p}-C_{s}\right)}{2\varsigma \omega_{n}R_{s}\left(C_{p}-C_{s}\right)+1}>0 \end{array}.$$

Then, we get

(49) $$C_{s}<C_{p}+\frac{\varsigma }{\omega_{n}R_{s}}+\frac{\theta^{2}}{2m\omega_{n}^{2}}-\frac{{\left\{{\left[\left(2\varsigma \omega_{n}\right)+\frac{\theta^{2}R_{s}}{m}\right]}^{2}-\left(\omega_{n}^{2}+\omega_{n}^{}\frac{\theta^{2}R_{s}}{2\varsigma m}\right)\right\}}^{0.5}}{2\omega_{n}^{2}R_{s}^{}}.$$

3. Negative inductance and negative capacitance in series with resistance 

When Γ*_R_* = 1, the characteristic function can be written as

(50) $$s^{2}\left(C_{p}-C_{s}+L_{s}C_{s}/m\right)+\left(2\varsigma \omega_{n}C_{p}-C_{s}-\theta^{2}R_{s}C_{s}/m\right)s+\omega_{n}^{2}\left(C_{p}-\theta^{2}/m-C_{s}\right)=0.$$

When *C_p_* < *C_s_*, according to the Routh–Hurwitz criterion, we have the following criterion:

(51) $$C_{s}<\min\left(C_{p}+L_{s}C_{s}/m,2\varsigma \omega_{n}C_{p}-\theta^{2}R_{s}C_{s}/m,C_{p}-\theta^{2}/m\right).$$

Therefore, *C_s_* should meet the following condition:

(52) $$C_{s}<C_{p}-\theta^{2}/m.$$

When *C_p_* > *C_s_*, with the same process, it can be found that

(53) $$C_{s}>C_{p}+L_{s}C_{s}/m.$$

#### 3.2.2. Root Locus Analysis

We already discussed the stability of the controlled stiffness system according to the Routh–Hurwitz criterion, where some assumptions and simplifications were made to obtain the final limitation expressions of *C_s_*. However, this cannot present the whole picture of the influence of shunt parameters. This section discusses the stability of the system with the root locus method.

1. Negative capacitance *C_s_*

This subsection analyzes the root locus of the piezoelectric stack transducer with respect to the negative capacitance *C_s_* for the three hybrid shunts. Firstly, the characteristic equation was written in form of the root locus form, allowing an easy simulation with MATLAB.

- Negative capacitance in series with resistance shunt:
  (54) $$1-\frac{C_{s}\left[mR_{s}C_{p}s^{3}+\left(m+R_{s}C_{p}c\right)s^{2}+\left(c+kR_{s}C_{p}+\theta^{2}R_{s}\right)s+k\right]}{mC_{p}s^{2}+cC_{p}s+\left(\theta^{2}+kC_{p}\right)}=0.$$
- Negative capacitance in parallel with resistance shunt:
  (55) $$1+\left(C_{p}-C_{s}\right)\frac{mR_{s}s^{3}+cR_{s}s^{2}+kR_{s}s}{ms^{2}+\left(c+\theta^{2}R_{s}\right)s+k}=0.$$
- Negative inductance/negative resistance in series with resistance shunt:
  (56) $$1+C_{s}\frac{L_{s}C_{p}ms^{4}+\left(L_{s}C_{p}c-R_{s}C_{p}m\right)s^{3}+\left(\theta^{2}L_{s}+L_{s}C_{p}k-m-R_{s}C_{p}c\right)s^{2}-\left(c+\theta^{2}R_{s}+R_{s}C_{p}k\right)s-k}{mC_{p}s^{2}+cC_{p}s+kC_{p}+\theta^{2}}=0.$$

**Figure 9:**
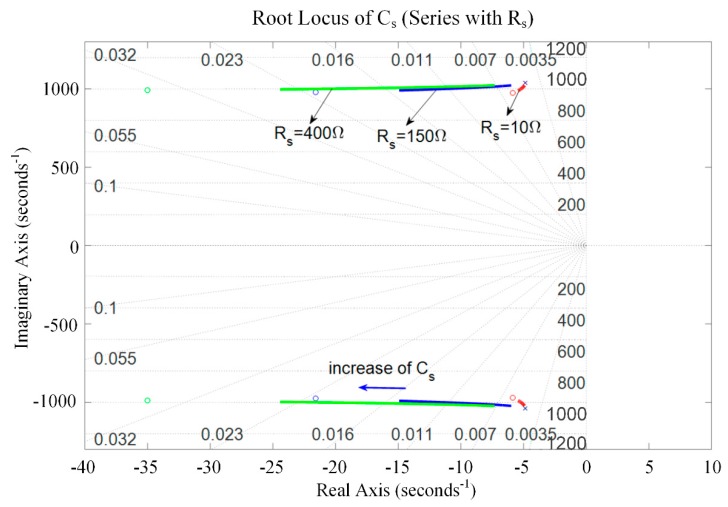
Root locus of the piezoelectric stack transducer with respect to *C_s_* for the negative capacitance in series with *R_s_*.

**Figure 10:**
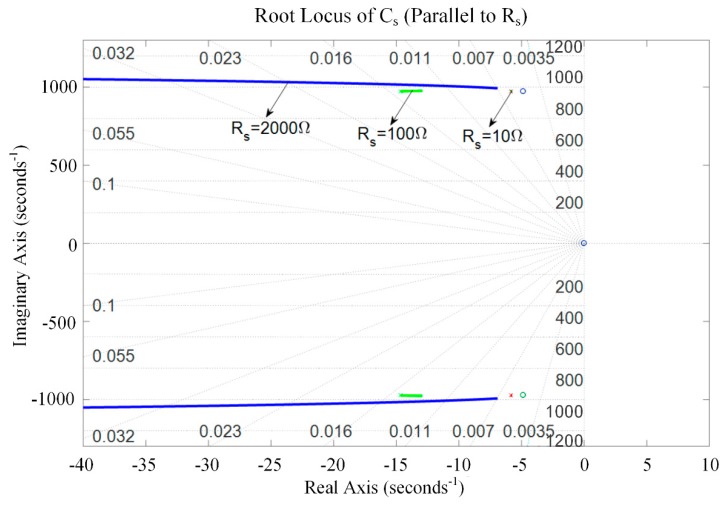
Root locus of the piezoelectric stack transducer with respect to *C_s_* for the negative capacitance in parallel with *R_s_*.

The root locus of the system with respect to *C_s_* was analyzed graphically to evaluate the stability of the system. Figure 9 and Figure 10 present the root locus of the piezoelectric stack transducer with respect to *C_s_* for the negative capacitance in series with *R_s_* and in parallel with *R_s_* cases, respectively. It can be found that the system is stable when *C_s_* is within [0.1, 5] μF. The damping improves with the increase of *R_s_*. An optimal *C_s_* can be found on the root locus curve. The results also imply that the negative capacitance in parallel with *R_s_* case has a relatively better damping performance than the negative capacitance in series with *R_s_* case. The hybrid negative-capacitance shunts can enhance stability when *C_s_* is selected carefully. 

**Figure 11:**
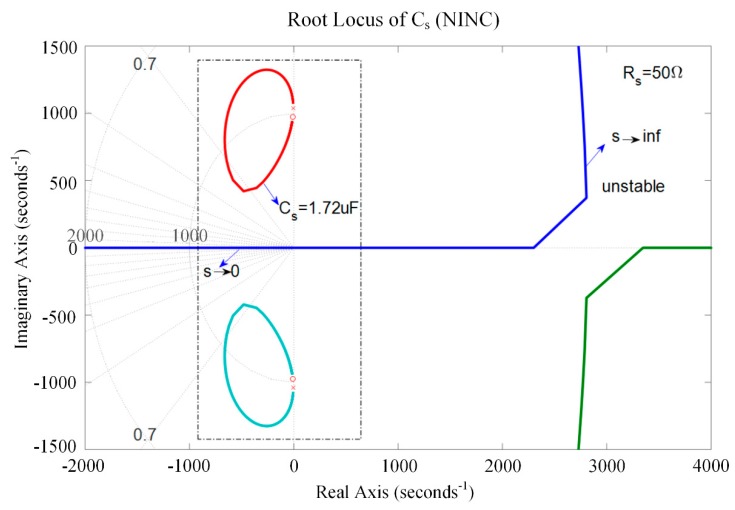
Root locus of the piezoelectric stack transducer with respect to *C*_s_ for the negative inductance/negative resistance in series with resistance shunt when *L_s_* = 10 mH and *R_s_* = 50 Ω.

Figure 11 presents the root locus of the piezoelectric stack transducer with respect to *C_s_* for the negative inductance/negative capacitance in series with resistance when *L_s_* = 10 mH and *R_s_* = 50 Ω. From Equation (56), it can be found that s → ∞ and *C_s_* → ∞ leads the system to be unstable. When s → 0, we have *C_s_* → ∞. Then, the root lies in the real axis. If *C_s_* is used carefully, the system can also be kept stable. In this case, relatively considerable damping can be achieved.

2. Adjustable Resistance *R_s_*

This subsection analyzes the root locus of the piezoelectric stack transducer with respect to the adjustable resistance *R_s_* for the three hybrid shunts. The characteristic equation is also written in the root locus form.

- Negative capacitance in series with resistance *R_s_*:
  (57) $$1-R_{s}\frac{mC_{s}C_{p}s^{3}+cC_{s}C_{p}s^{2}+\left(\theta^{2}C_{s}+kC_{s}C_{p}\right)s}{m\left(C_{p}-C_{s}\right)s^{2}+c\left(C_{p}-C_{s}\right)s+\theta^{2}+\left(C_{p}-C_{s}\right)k}=0.$$
- Negative capacitance in parallel with resistance *R_s_*:
  (58) $$1+R_{s}\frac{m\left(C_{p}-C_{s}\right)s^{3}+c\left(C_{p}-C_{s}\right)s^{2}+\left[\theta^{2}+k\left(C_{p}-C_{s}\right)\right]s}{ms^{2}+cs+k}=0.$$
- Negative inductance/negative resistance in series with resistance *R_s_*:
  (59) $$1-\frac{R_{s}\left(C_{p}C_{s}ms^{3}+C_{p}C_{s}cs^{2}+\left(\theta^{2}+C_{p}k\right)C_{s}s\right)}{L_{s}C_{p}C_{s}ms^{4}+L_{s}C_{p}C_{s}cs^{3}+\left(\theta^{2}L_{s}C_{s}+L_{s}C_{s}C_{p}k+mC_{p}-C_{s}m\right)s^{2}+\left(C_{p}-C_{s}\right)cs+\left(C_{p}-C_{s}\right)k+\theta^{2}}=0.$$

**Figure 12:**
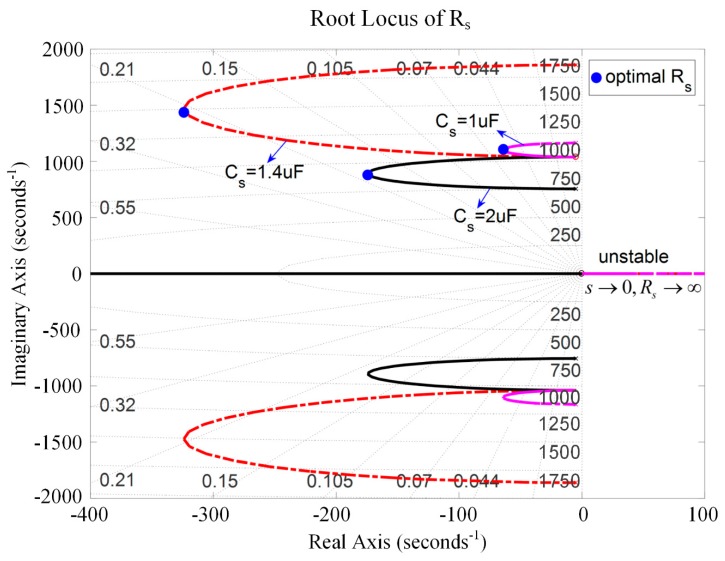
Root locus of the piezoelectric stack transducer with respect to *R_s_* for the negative capacitance in series with *R_s_*.

**Figure 13:**
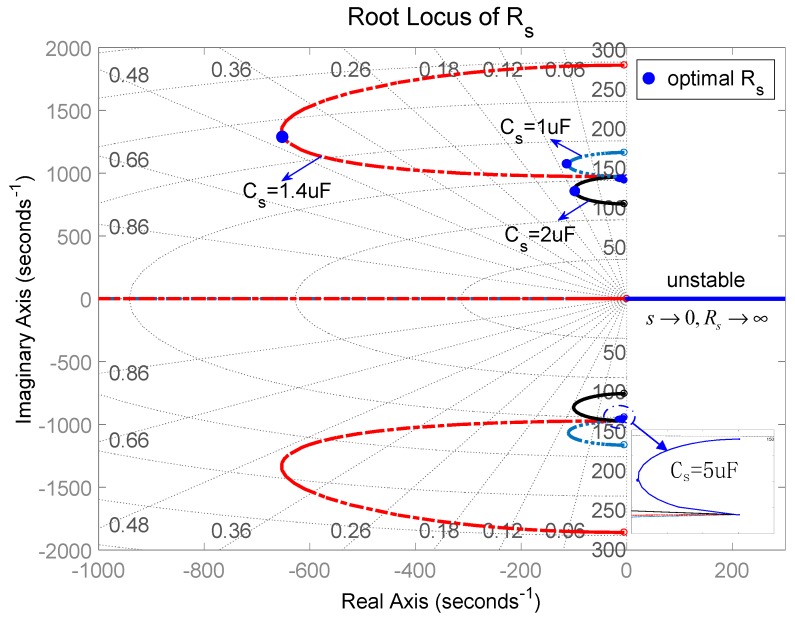
Root locus of the piezoelectric stack transducer with respect to *R_s_* for the negative capacitance in parallel with *R_s_*.

**Figure 14:**
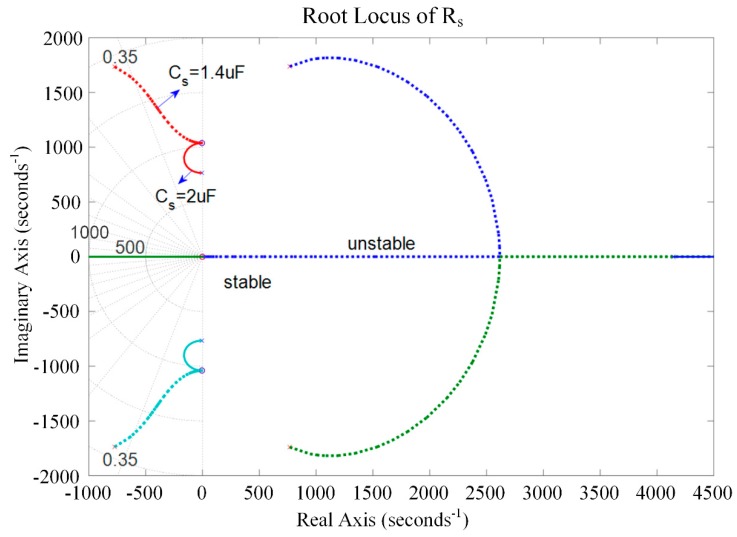
Root locus of the piezoelectric stack transducer with respect to *R_s_* for the negative inductance/negative resistance in series with *R_s_*.

Equations (57) and (58) demonstrate that *s* → ∞ results in *R_s_* → ∞. The root lies in the real axis. Figure 12 and Figure 13 show the root locus of the system with respect to *R_s_* for negative capacitance in series with *R_s_* and in parallel with *R_s_*, respectively. The results prove the correctness of the theoretical model. In this case, some roots are positive, which makes the system unstable. In other ranges, the system can maintain stability with the change of *C_s_* (*C_s_* = 1 μF, 1.4 μF, and 2 μF). When *C_s_* = 1.4 μF, we get a considerable damping performance, and the corresponding optimal *R_s_* can also be found in Figure 12 and Figure 13. Moreover, the parallel *R_s_* case has relatively good damping performance compared to the series *R_s_* case. Figure 14 is the root locus of the system with respect to the negative inductance/negative capacitance when *C_s_* = 1.4 μF and 2 μF. The result shows that the system is conditionally stable with the change of *R_s_*. One should carefully choose *R_s_*, *C_s_*, and *L_s_*.

### 3.3. Frequency Response Analysis

#### 3.3.1. Piezoelectric Shunt Stiffness

As shown in the theoretical analysis of the PSS and PSD effects in the negative-impedance shunted piezoelectric stack transducer, all three hybrid shunts can achieve the controlled stiffness performance. The frequency response of the system was determined in order to further discuss the influence of PSS and PSD on the vibration control performance. 

**Figure 15:**
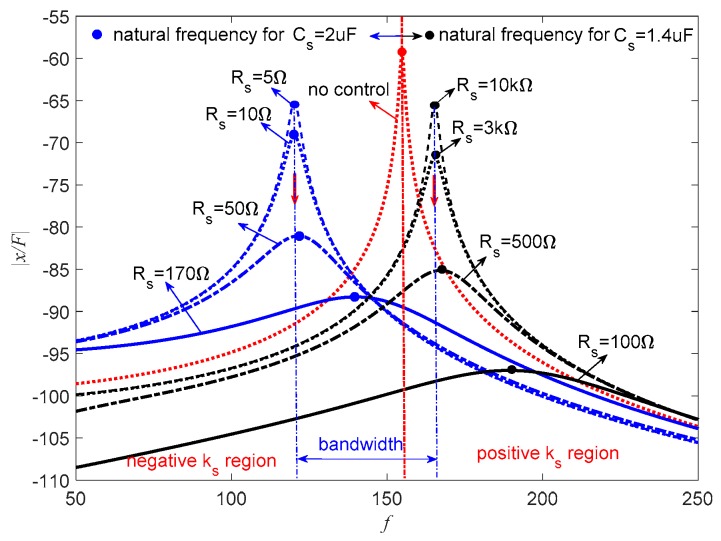
Frequency response of the piezoelectric stack transducer with negative capacitance in series with *R_s_*.

Figure 15 represents the frequency response of the piezoelectric stack transducer for the negative capacitance in series with *R_s_* case. It can be found that the PSS is positive, which increases the natural frequency of the transducer when *C_s_* = 1.4 μF. When *R_s_* = 10 kΩ, the amplitude approximates to the uncontrolled condition. With the decrease of *R_s_*, the amplitude decreases while the natural frequency increases. When *C_s_* = 2 μF, this hybrid shunt can produce the negative stiffness effect, and the natural frequency of the system also decreases. In this case, the amplitude decreases with the increase of *R_s_*. The corresponding optimal *R_s_* can be found from Figure 12. The damping performance of PSD is shown in Table 2; it can be seen that PSD can achieve wonderful damping performance compared with the traditional pure resistive shunt method. 

**Figure 16:**
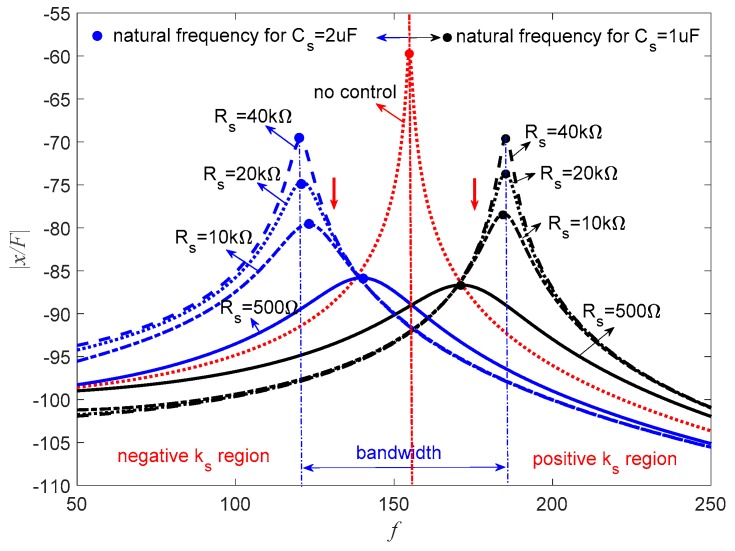
Frequency response of the piezoelectric stack transducer with negative capacitance in parallel with *R_s_*.

As suggested in Figure 7, the PSS is sensitive to *R_s_* when *C_s_* = 1.4 μF. *C_s_* = 1 μF is a better choice. Figure 16 shows the corresponding frequency response of the piezoelectric stack transducer in parallel with *R_s_* with the change of *C_s_* and *R_s_*. When *C_s_* = 1 μF, the natural frequency increases, which means PSS is positive for *C_p_* > *C_s_*, and the amplitude decreases with the decrease of *R_s_*. When *C_s_* = 2 μF, the natural frequency decreases, which indicates that PSS is negative for *C_p_* < *C_s_*, and the amplitude decreases with the increase of *R_s_*. The amplitude of the transducer can be controlled by the change of *R_s_*.

**Figure 17:**
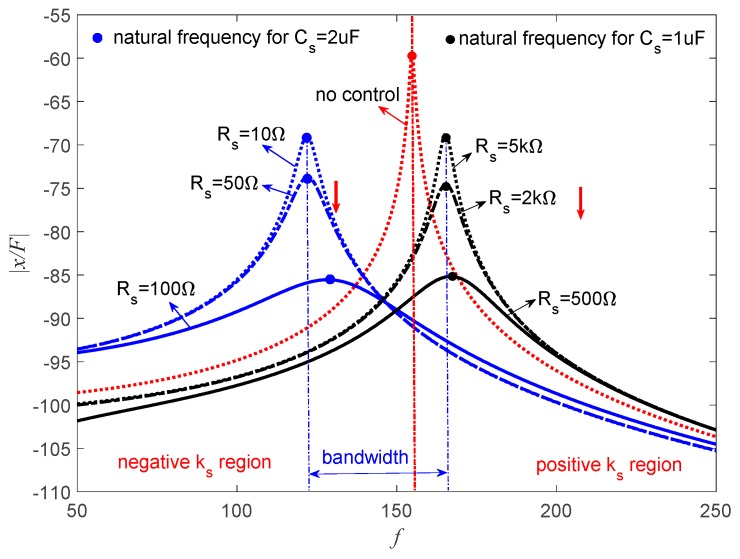
Frequency response of the piezoelectric stack transducer with negative inductance/negative capacitance in series with *R_s_* when *L_s_* = 10 mH.

Figure 17 represents the frequency response of the piezoelectric stack transducer with negative inductance/negative capacitance in series with *R_s_* when *L_s_* = 10 mH. When *C_s_* = 1 μF, the natural frequency increases, which means the PSS is positive. The amplitude decreases with the decrease of *R_s_*. While *C_s_* = 2 μF, the natural frequency decreases, which means the PSS is negative, and the amplitude decreases with the increase of *R_s_*. 

This implies that *R_s_* can be carefully selected to increase the damping of the system without changing the stiffness of the system, which is important in some special applications. Figure 15, Figure 16 and Figure 17 also indicate that the bandwidth performance of the negative capacitance in parallel with *R_s_* shunt is better than the other two cases, which can provide considerable controlled stiffness performance. If this transducer is used as an isolator, negative stiffness is a better choice. If one just wants to avoid the resonance of the system, both positive and negative stiffness are acceptable. The previous study by Heuss et al. [52] utilized different combinations of resistant, resonant, and negative capacitance to achieve the tuning of a vibration absorber. The tuning frequency band can be 120 Hz. We can also achieve this performance if negative capacitance and adjusting resistance are carefully designed. 

#### 3.3.2. Low-Frequency Vibration Control

**Figure 18:**
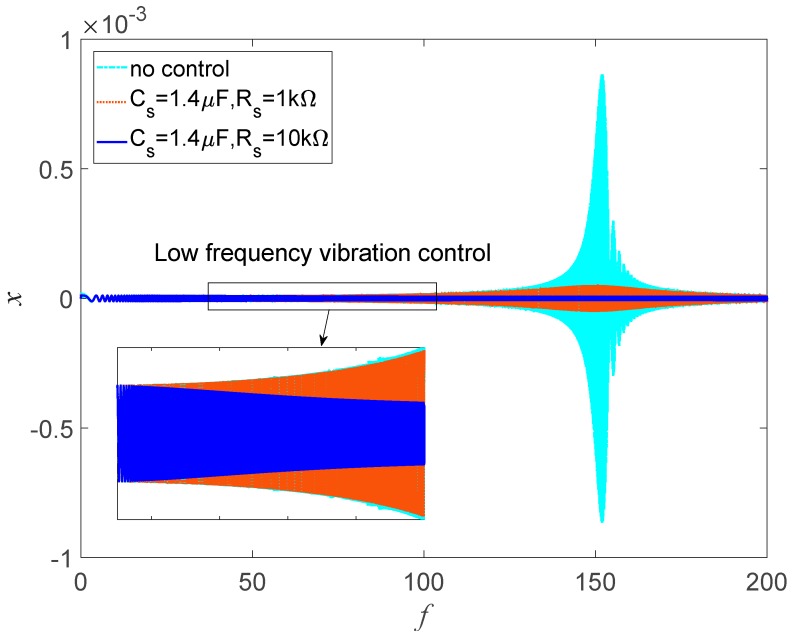
Time history response of the transducer under sweep sine excitation.

Figure 18 shows the time history response of the transducer under sweep sine excitation when *Cs* is 1.4 μF. When *R_s_* is 1 kΩ, only the response near the resonance is controlled. When *R_s_* increases to 10 kΩ, the natural frequency increases. The response decreases dramatically near the resonance. Furthermore, the low-frequency vibration is also suppressed, and the bandwidth can reach up to 150 Hz. In view of vibration isolation, low-natural-frequency isolators can achieve bandwidth isolation performance when the excitation frequency is bigger than $\sqrt{2}\omega_{n}$, such as nonlinear vibration isolators [56,57,58], quasi-zero isolators [59,60], etc. These nonlinear vibration isolators can achieve broadband vibration isolation performance with negative dynamic stiffness of nonlinear isolators; however, the vibration suppression in the resonance region is dependent on damping. The proposed PSS can semi-actively decrease the stiffness of linear isolators to improve the vibration performance; therefore, it has application potential in isolation engineering. Furthermore, the PSS can also increase the stiffness of isolators to enhance the vibration suppression performance in the resonance region. 

## 4. Conclusions

In this study, we proposed the novel controlled stiffness performance of a rhombic piezoelectric stack transducer with hybrid negative-impedance shunts. The governing equation of the transducer was established according to Lagrange’s equation. Piezoelectric shunt stiffness and piezoelectric shunt damping were defined to analyze the stiffness and damping effects of transducer with three kinds of hybrid shunts. The Routh–Hurwitz criterion was employed to get the theoretical selection of negative capacitance. The root locus method was utilized to graphically judge the stability of the proposed three kinds of hybrid shunts. The results demonstrate that the piezoelectric stack transducer can produce both the stiffness and damping effects with hybrid shunts. With the change of negative capacitance, both negative and positive stiffness can also be obtained. Moreover, the negative stiffness effect requires a careful choice of the negative capacitance to sustain the stability of the system. Furthermore, negative capacitance in parallel with resistance demonstrated a considerably better stiffness bandwidth and damping performance than the other two shunts. The proposed PSS can be used to decrease the stiffness to decrease the natural frequency and, thus, to increase the vibration isolation band of linear or nonlinear isolators. Additionally, the PSS can be also used to adjust the stiffness to avoid resonance when the host structure is subjected to harmonic excitations. Future research may focus on experimental investigations of the PSS.

## Figures and Tables

**Table 1:** Parameters of the piezoelectric stack and the hybrid shunts.

Parameters (Unit)	Value
Piezoelectric charge coefficient, *d*_33_ (C/N)	400 × 10^−12^
Capacitance of the stack, *C_p_* (μF)	1.478
*R* (Ω)	1 × 10^6^
*T_R_*	1
*L_s_* (mH)	10
Mass, *m* (kg)	0.1
Natural frequency of transducer, *f_n_* (Hz)	154.9

**Table 2:** Comparison of damping ratio shown in Figure 15.

Negative Capacitance	Adjustable Resistance	Damping Ratio, ζ
Resistive load	*R_s_* = 100 Ω	0.005
*C_s_* = 2 μF	*R_s_* = 5 Ω	0.0706
	*R_s_* = 10 Ω	0.0973
	*R_s_* = 50 Ω	0.2553
	*R_s_* = 170 Ω	0.4721
*C_s_* = 1.4 μF	*R_s_* = 10 kΩ	0.0381
	*R_s_* = 3 kΩ	0.0648
	*R_s_* = 500 Ω	0.1684
	*R_s_* = 100 Ω	0.26
